# Spectrum of HIV-associated infectious diseases: A case series through the eyes of the histopathologist

**DOI:** 10.4102/sajhivmed.v21i1.1087

**Published:** 2020-06-29

**Authors:** Reena Mohanlal, Denasha L. Reddy

**Affiliations:** 1 Department of Anatomical Pathology, Faculty of Health Sciences, University of the Witwatersrand, Johannesburg, South Africa; 2 National Health Laboratory Services, Chris Hani Baragwanath Academic Hospital, Johannesburg, South Africa; 3 Division of Infectious Diseases, Department of Internal Medicine, Chris Hani Baragwanath Academic Hospital, University of the Witwatersrand, Johannesburg, South Africa

**Keywords:** infectious diseases, histology, HIV, opportunistic infections, diagnosis

## Abstract

**Background:**

Human immunodeficiency virus (HIV) infection increases the risk of infection by a host of other opportunistic pathogens. The clinical presentations of these co-infections in immunocompromised patients are often atypical; therefore diagnosis is delayed in the absence of investigations such as tissue biopsy. Infection may involve sites that are difficult to access for biopsy and, as a consequence, there is limited diagnostic tissue available for analysis. The histopathologist, aided by ancillary tests, is relied upon to make a timeous and accurate diagnosis.

**Objectives:**

To illustrate key histological features of HIV-associated infectious diseases encountered in a histopathology laboratory and to highlight, with the aid of literature, the relevance of histopathology in diagnosis.

**Method:**

A retrospective descriptive case series of biopsies histologically diagnosed with HIV-associated infectious diseases over four years (2015–2019) was performed at the Chris Hani Baragwanath Academic Hospital National Health Laboratory Services Histopathology department. These cases have been photographed to illustrate microscopic aspects and will be accompanied by a literature review of opportunistic infections in the context of HIV infection.

**Results:**

This article highlights aspects of fungal, parasitic, viral and selected bacterial infections of people living with HIV for whom the histopathological examination of tissue was an essential component of the clinical diagnosis. Histological features are noted on routine slides and accompanied by diagnostic features revealed with histochemical and immunohistochemical stains.

**Conclusion:**

Medical practitioners working in areas of high HIV endemicity should be familiar with the variety of infectious diseases that are encountered and with the diagnostic importance of the histopathologist in clinical management.

## Introduction

People living with human immunodeficiency virus (HIV) are at risk from multiple infective pathogens. The clinical presentation of these infections is often atypical. This can result in costly diagnostic and therapeutic delays. Adequate tissue is sometimes inaccessible for re-biopsy, is of limited quantity, or may have been fixed in formalin and is therefore useless for routine microbiological culture. What the histopathologist has may be all the material there is. In the absence of a confirmatory microbiological ‘answer’, the histopathologist must maintain a high index of suspicion for infectious diseases, perform special stains and exclude multiple pathogens even after one has been identified.^1^ There is limited exposure to histopathology in undergraduate medical training in South Africa (SA) and clinicopathological meetings for postgraduate teaching are mostly confined to large academic centres. This article highlights aspects of fungal, parasitic, viral and selected bacterial infections encountered in the context of HIV infection and immunosuppression. A series of cases diagnosed with the aid of the histopathology laboratory of the National Health Laboratory Service (NHLS) of the Chris Hani Baragwanath Academic Hospital (CHBAH) in Soweto, SA, has been collected to demonstrate the importance of the histopathologist to the HIV clinician and infectious diseases specialist.

## Fungal infections

### Case 1

**FIGURE 1:**
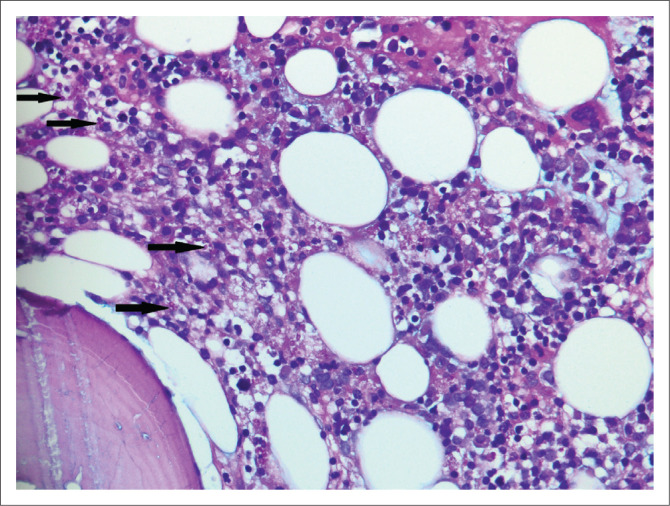
Bone marrow trephine biopsy with intracellular fungal yeasts (arrow) that are Periodic Acid Schiff positive with Alcian Blue Periodic Acid Schiff stain (original magnification × 400).

A bone marrow trephine biopsy from an HIV-positive man with bi-cytopaenia was received. Intracellular fungal yeasts were noted on special stains (Figure 1). A diagnosis of fungal infection was made and correlation with fungal culture was advised.

### Case 2

**FIGURE 2:**
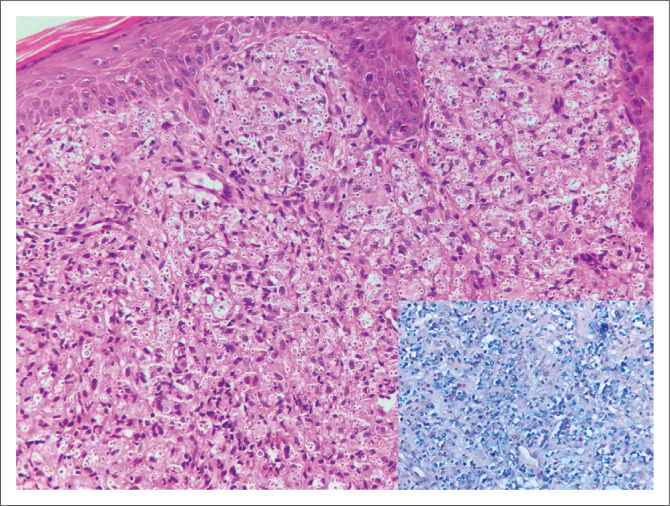
Skin biopsy with numerous extracellular yeasts on haematoxylin and eosin (original magnification × 200), the inset shows the yeasts with blue capsules and pink cell walls on Alcian Blue Periodic Acid Schiff stain (original magnification × 400).

A skin punch biopsy was submitted from a 32-year-old woman with umbilicated skin lesions. Cryptococcal latex antigen on serum and cerebrospinal fluid (CSF) were positive and CSF fungal culture revealed *Cryptococcus neoformans*. Her CD4 count was 1 cell/µL. Numerous extracellular yeasts were seen on haematoxylin and eosin stained section (Figure 2). The capsules and cell walls were highlighted on Alcian Blue Periodic Acid Schiff (ABPAS) special stain (Figure 2, inset) and the diagnosis of cryptococcosis was made.

**Comment:** The histopathologist is often relied upon to make a timeous diagnosis of a fungal infection as tissue may not have been submitted for fungal culture or an ‘extended’ culture may be required, leading to a longer diagnostic turnaround time. Although fungal culture remains the gold standard, the diagnosis of a fungal infection can be reliably made on histological examination by identifying fungal yeasts (see Figure 1) or hyphae using histochemical stains such as Grocott and Periodic Acid Schiff (PAS). Histological features of fungal infection that should prompt special staining for fungal elements include granulomatous inflammation, neutrophilic micro-abscesses, foamy histiocytes, ulceration, pseudoepitheliomatous hyperplasia and suppurative inflammation.^2^ Some morphological clues may point towards a specific fungus; for example *Cryptococcus* yeasts, which are extracellular and variably sized. The typical staining on ABPAS special stain, as was noted in case 2 (see Figure 2), is supportive of the diagnosis. *Pneumocystis jirovecii* is typically present within a foamy exudate, the organisms are Grocott positive and appear as collapsed ‘helmets’ with a central dot. In a South African cohort of patients from the Western Cape, emergomycosis (previously emmonsiosis) was the most common systemic mycoses, followed by sporotrichosis and histoplasmosis.^3^ It is not possible to distinguish emergomycosis from other fungi on histological examination.^4^ Serum β-D-glucan and urine *Histoplasma capsulatum* antigen testing can be used as adjuncts when a fungal infection is clinically suspected. It is worthwhile remembering that urine *H. capsulatum* antigen can be positive in patients with emergomycosis due to cross-reactivity.^3,4^ Once the histological diagnosis of a fungal infection is made, further material should be submitted for fungal culture or polymerase chain reaction (PCR). Although further confirmatory investigations were not performed in case 1, definitive identification of fungal species by these methods is critical as they will impact the choice and duration of antifungal therapy.

## Bacterial infections

### Mycobacterial infection

#### Case 3

**FIGURE 3:**
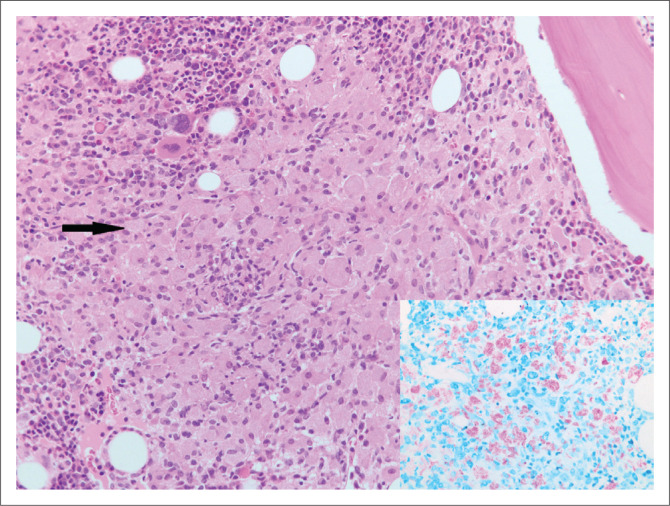
Bone marrow trephine biopsy showing histiocytes (arrows) (original magnification × 200) with acid fast bacilli in clumps on Ziehl Neelsen stain (inset) (original magnification × 400).

A 40-year-old HIV-positive woman had bi-cytopaenia on full blood count. Histological examination of the bone marrow trephine biopsy showed an infiltrate of foamy histiocytes. Numerous, clumped intracellular acid-fast bacilli were noted on Ziehl Neelsen (ZN) stain (Figure 3). Culture yielded growth of a non-tuberculous mycobacterium and PCR confirmed *Mycobacterium avium complex.*

**Comment:** The synergy between the *Mycobacterium* species and HIV is well documented. Patients with HIV have progressive and disseminated mycobacterial diseases, and, in turn, mycobacterial infection increases HIV replication.^5^ The histological presentations of mycobacterial infection are varied. The prototypic feature noted on microscopic examination is granulomatous inflammation. However, with advanced immunosuppression, granulomas are usually absent and neutrophilic infiltration and necrosis are prominent.^6^ Mycobacterial spindle cell pseudotumour is another manifestation of mycobacterial infection seen more commonly in lymph node biopsies. This entity is characterised by a proliferation of spindled histiocytes and fibroblasts and positive ZN stain. It may mimic a host of mesenchymal tumours due to the spindled appearance of the cells, thus leading to misdiagnosis.^6,7^ Bacille Calmette-Guérin (BCG) infection may manifest as regional (BCGitis) or systemic disease (BCGosis) following BCG vaccination.^8^ In addition, BCGitis may occur after commencement of antiretroviral therapy (ART) as part of immune reconstitution. This should be borne in mind, especially when children present with lymphadenitis involving axillary or supraclavicular nodes and granulomatous inflammation is noted on histological examination.^8,9^ Testing for *Mycobacterium bovis* is indicated in this setting.^8^ Tuberculids such as erythema induratum are hypersensitivity reactions to mycobacterial antigens and no acid-fast bacilli are demonstrated in tissue biopsies from these lesions.^10^ Although definitive mycobacterial species identification is not possible on histological examination, the finding of sheets of foamy histiocytes containing ZN and PAS-positive bacilli are suggestive of *Mycobacterium avium complex* as noted in case 3 (see Figure 3). Acid-fast bacilli with a long beaded appearance may indicate infection with *Mycobacterium kansasii*.^5^ In cases where granulomatous inflammation morphologically in keeping with mycobacterial infection or acid-fast bacilli are noted on histological examination, samples should be submitted for mycobacterial culture and sensitivity, and when appropriate – molecular testing. It should be noted that there may be false positives on ZN stain due to laboratory contamination that occurs during preparation. Histopathologists are always sensitised to this possibility and contaminants are recognised as such as they lie on a different plane, are clumped or are free-lying, away from the tissue section.^5^

### Bartonella infection

#### Case 4

A 34-year-old man on ART presented with a fungating mass on the right foot. He had a CD4 count of 21 cells/µL. Features of bacillary angiomatosis were noted on routinely stained skin biopsy and Warthin-Starry stain confirmed the presence of bacilli; PCR was not requested.

**FIGURE 4:**
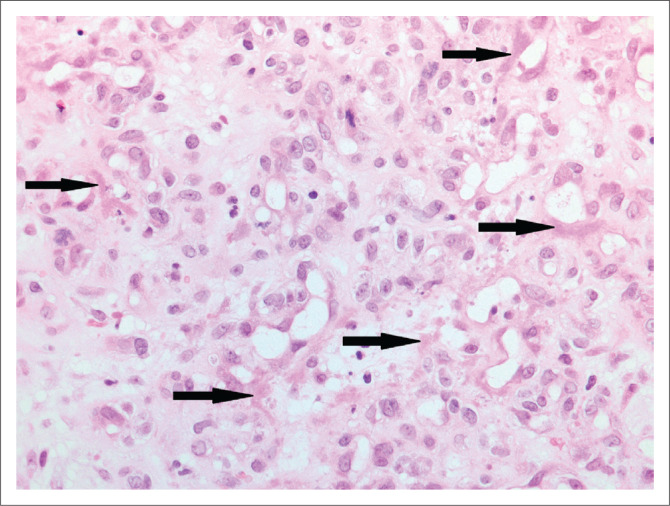
A proliferation of capillaries is noted with basophilic clumps of bacteria, indicated by arrows, characteristic of bacillary angiomatosis (original magnification × 400).

Comment: Histologically, bacillary angiomatosis comprises a lobular proliferation of vascular spaces with plump endothelial cells and neutrophilic debris.^11^ The bacilli in bacillary angiomatosis are noted as basophilic clumps in the tissue stroma where they proliferate (Figure 4). The bacteria induce endothelial anti-apoptosis and a pro-inflammatory state which accounts for the histological features that are noted.^12^ Warthin-Starry special stain or PCR for Bartonella genus can be used to confirm the diagnosis. The microscopic differential diagnosis includes a pyogenic granuloma or Kaposi sarcoma. While Bartonella infection manifests most commonly as bacillary angiomatosis in immunocompromised patients, it may also cause peliosis in the liver, endocarditis, osteomyelitis and cat-scratch disease.^12^

### Syphilis

#### Case 5

**FIGURE 5:**
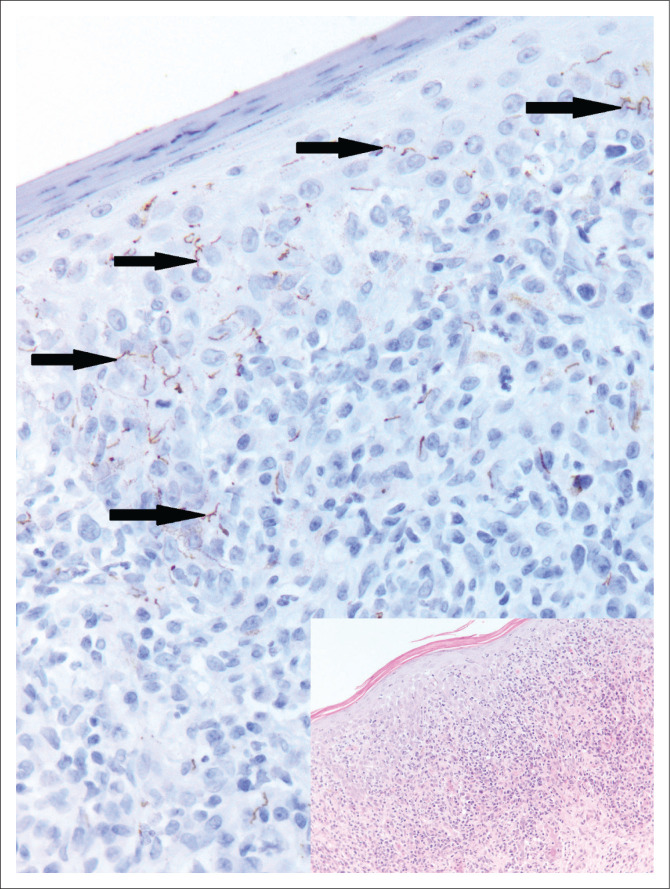
Numerous spirochaetes are identified on *T. pallidum* immunohistochemistry performed on the skin biopsy (original magnification × 400). A lichenoid lymphoplasmacytic infiltrate is noted on haematoxylin and eosin (inset) (original magnification × 200).

A 32-year-old woman who was recently commenced on ART presented to the dermatology clinic with a generalised maculopapular rash involving her palms and soles. Her CD4 count was 219 cells/µL. A lichenoid lymphoplasmacytic infiltrate was noted on haematoxylin and eosin stained section (Figure 5, inset). Numerous spirochaetes were identified on *Treponema pallidum* immunohistochemistry, confirming the diagnosis of secondary syphilis (Figure 5). Serum *T. pallidum* antibody was positive and rapid plasma reagin test was reactive with a titer of 1024.

**Comment:** The prevalence of syphilis is increasing. In Canada, the United States and the United Kingdom, an increasing incidence is noted in men who have sex with men. Skin lesions are the most amenable to biopsy in suspected syphilis. Papulosquamous lesions with palmo-plantar involvement are the typical clinical findings but atypical presentations such as alopecia, pustular lesions, annular rash and nodules have also been described. Patients with HIV infection are more likely to have atypical presentations and secondary syphilis at the time of diagnosis.^13^ Histopathological findings in secondary syphilis include epidermal hyperplasia and a moderate to dense lymphoplasmacytic infiltrate as were noted in case 5 (Figure 5, inset). The *T. pallidum* immunohistochemical stain (Figure 5) is more sensitive than a silver stain. The sensitivity of immunohistochemistry was 64% compared to 9% for the silver stain, in detecting spirochaetes in one study. The same study also showed that patients with CD4 counts less than 250 cells/mL had more organisms (> 100 treponemes in 10 high power fields) demonstrated on skin biopsy than those with CD4 counts above 250 cells/mL.^14^

## Viral infections

### Case 6

**FIGURE 6:**
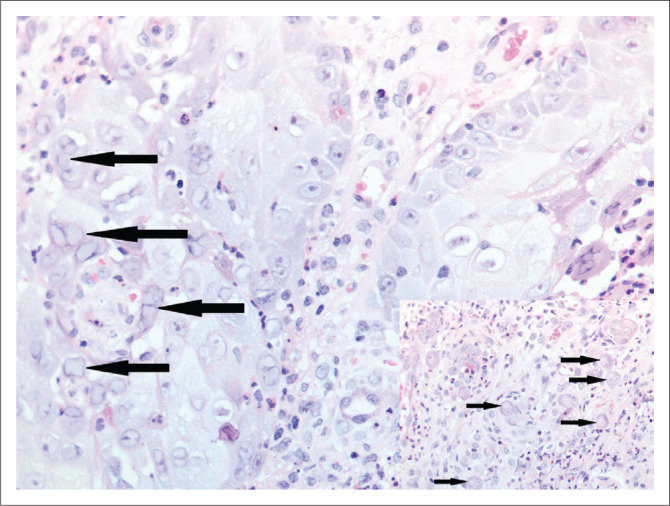
Perianal biopsy showing herpes simplex virus and Cytomegalovirus inclusions (inset) in the same biopsy (both original magnification × 400).

A 32-year-old woman presented with a one-month history of a perianal lesion. She had a CD4 count of 18 cells/µL. Herpes simplex virus (HSV) and Cytomegalovirus (CMV) inclusions were both present in the perianal biopsy (Figure 6). No serological testing for HSV or CMV were performed.

#### Comment

Infection with HSV2 typically presents as genital ulcers. There is a strong association between HIV and HSV2 infection.^15^ Individuals infected with HSV2 have sixfold higher odds of HIV infection compared with those uninfected with HSV2, and 68% of patients with genital ulcers caused by HSV2 were found to be co-infected with HIV. Locally, HSV2 remains the leading cause of pathogen detectable genital ulcer disease.^16^ Histologically, cells infected with HSV2 show intranuclear inclusions with a glassy appearance, margination of chromatin and multinucleation. Infected keratinocytes are best demonstrated at the ulcer edge (Figure 6). However, in hypertrophic or tumourous HSV2 lesions, the dense inflammatory cell infiltrate may obscure the viral inclusions. Careful search of multiple levels through the tissue block and immunohistochemistry may be required to identify the virally infected cells. Sparse infected cells within the deep dermis derived from ruptured hair follicles may be seen. It has been postulated that the florid inflammation noted in these hypertrophic lesions is due to immune reconstitution.^17^ Tissue may be submitted for drug resistance testing in those cases not showing clinical response to standard therapy. Cells infected with HSV and varicella-zoster virus show identical features on histological examination. Immunohistochemical stains are available to help distinguish among them.

Characteristic intracytoplasmic and intranuclear CMV inclusions were also seen in case 6 (Figure 6, inset) but atypical histological features are well documented.^18^ Immunohistochemistry can also be used in those cases that are densely inflamed. The role of CMV that is detected in mucocutaneous lesions is controversial. It is thought by some that the virus does not cause the lesion but is merely a bystander and signifies that there is generalised CMV infection. Its presence in genito-anal lesions may be as a result of autoinoculation of virus shed in faeces. Possible re-activation of latent virus in endothelial cells or haematogenous spread of the virus to granulation tissue is also postulated.^19^ Cytomegalovirus gastrointestinal tract disease can manifest as ulceration or polyps endoscopically and can show co-infection with other organisms such as *Cryptosporidium*.^20^ Viral load testing for CMV may be useful in confirming disease and in monitoring treatment response.

### Case 7

**FIGURE 7:**
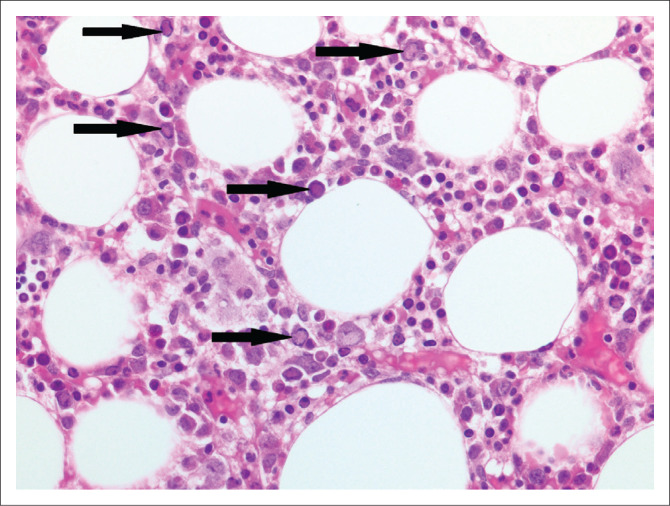
Bone marrow trephine biopsy with numerous parvovirus inclusions (arrows) (original magnification × 400).

A bone marrow trephine biopsy was submitted from a 29-year-old HIV-positive woman known to have pulmonary tuberculosis and a CD4 count of 80 cells/µL. Her red cell count was 2.31 × 10^12^/L, her reticulocyte count was 7.41% and her haemoglobin was 7.0 g/dL. Numerous parvovirus inclusions were noted on microscopic examination (Figure 7).

#### Comment

Parvovirus B19 infection of the bone marrow can manifest as a transient aplastic crisis or persistent infection with pure red cell aplasia. On microscopic examination of a bone marrow trephine biopsy with parvovirus infection, erythroid precursors are absent and giant pro-normoblasts^21^ are seen (Figure 7). Morphologically suspicious cases can be confirmed with immunohistochemistry. While PCR testing for parvovirus B19 is very sensitive, detection of parvovirus B19 DNA in the blood does not equate to acute infection.^22^ Parvovirus B19 DNA has also been detected in asymptomatic, parvovirus B19 IgM negative individuals in solid organs such as skin, myocardium, synovium and bone marrow.^23^ No PCR or viral load testing was performed in our case.

## Helminthic infections

### Case 8

**FIGURE 8:**
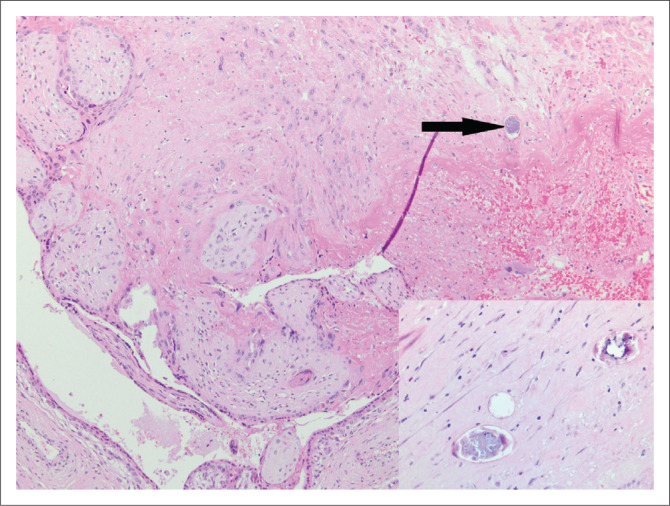
Fallopian tube with products of conception and an incidental finding of schistosomal ova (arrow) (original magnification × 100) and inset (original magnification × 400).

A fallopian tube was excised for an ectopic pregnancy and submitted for histology. Schistosomal ova were noted incidentally within the fallopian tube on microscopic examination (Figure 8).

#### Comment

Schistosomiasis is diagnosed on histology in biopsy specimens from the urinary bladder, cervix, fallopian tube, appendix, liver and colon. The ova are elliptical in shape and may be calcified (Figure 8). A *Schistosoma haematobium* ovum has a terminal spine, while a *Schistosoma mansoni* ovum has a lateral spine. This distinguishing feature is difficult to apply in histology due to variability in the plane of sectioning. *Schistosoma mansoni* ova are positive with a ZN stain while *S. haematobium* ova are negative. The ova elicit a granulomatous or eosinophilic inflammatory response. Haemazoin pigment, a fine black non-refractile pigment, is another useful clue to the presence of schistosomiasis. It is formed by digestion of haemoglobin present in the worm gut following ingestion of red blood cells.^24^ Schistosomiasis is thought to increase susceptibility to HIV infection by disrupting the mucosal barrier and increasing vascularisation and recruitment of CD4 positive T cells.^25^ Although treatment kills the adult worms, the lesions may persist. Tissue diagnosis in female genital schistosomiasis is crucial as it is thought to impact fertility.^26^ Stool and urine microscopy are the gold standard to assess for infection but suffer from a lack of sensitivity as the eggs are not always detectable in the urine.^27^ Bladder biopsies are usually submitted to the laboratory with the clinical information of the characteristic ‘sandy patches’ appearance noted on cystoscopy. In other sites, however, the diagnosis is often rendered incidentally in biopsies submitted for other pathologies such as in case 8.

Other helminthic infections that can be readily diagnosed by the histopathologist are neurocysticercosis and hydatid disease. HIV-positive individuals with neurocysticercosis may present with multiple parenchymal lesions and co-infection with HIV has been reported in almost a third of cases of neurocysticercosis.^28^ On microscopic examination, the cysts have three layers, including outer cuticular, middle cellular and inner reticular layers.^29^ Hydatid disease is caused by species of the *Echinococcus* genus.^30^ Histologically proven cases of hydatid disease have increased and may be due to the increase in HIV prevalence. Lungs and liver are the more commonly affected organs.^31^ The cyst wall appears thin, smooth and white macroscopically, and on histological examination appears eosinophilic, acellular and lamellar. Cytological examination of the cyst fluid shows refractile hooklets and scolices.^30^ While HIV is a risk factor for infection with *Strongyloides stercoralis*, hyperinfection with autoinfection and disseminated disease that is associated with immunosuppression are rarely reported in HIV-positive patients.^32^ The histopathologist plays a limited role in the diagnosis of strongyloidiasis as stool samples are usually submitted for confirmation. An eosinophilic infiltrate with microabscess formation and degranulation on histological examination of mesenteric lymph nodes may be a clue to the presence of strongyloides infection.^33^

## Conclusion

Histopathology plays an important role in the diagnosis of infectious diseases as many have characteristic histological features. Pathologists practising in areas with a high HIV prevalence are attuned to the importance of investigating inflamed tissue biopsies for an underlying infectious agent with special and immunohistochemical stains and liaising with clinicians to initiate timeous therapy. Correlation with relevant investigations such as PCR, culture and sensitivity, is advised in all cases to inform treatment decisions. With the aid of this case series and accompanying histology images, some key features of infections encountered in practice are conveyed to demonstrate the relevance of histopathology.

## References

[1] Grayson W Recognition of dual or multiple pathology in skin biopsies from patients with HIV/AIDs. Patholog Res Int. 2011;2011:398546 10.4061/2011/398546 21789262 PMC3135116

[2] Fernandez-Flores A, Saeb-Lima M, Arenas-Guzman R Morphological findings of deep cutaneous fungal infections. Am J Dermatopathol. 2014;36(7):531–556. 10.1097/DAD.0b013e31829cc6f3 24950417

[3] Schwartz I, Kenyon C, Lehloenya R, et al AID-related endemic mycoses in Western Cape, South Africa and clinical mimics: A cross sectional study of adults with advanced HIV and recent onset widespread skin lesions. Open Forum Infect Dis. 2017;4(4):1–7. 10.1093/ofid/ofx186 PMC5695619 29164168

[4] Schwartz I, Govender N, Corcoran C, et al Clinical characteristic, diagnosis, management and outcomes of disseminated emmonsiosis: A retrospective case series. Clin Infect Dis. 2015;61(6):1004–1012. 10.1093/cid/civ439 26060283

[5] Procop G HIV and mycobacteria. Semin Diagn Pathol. 2017;34(4):332–339. 10.1053/j.semdp.2017.04.006 28550962

[6] Michelow P, Omar T, Field A, Wright C The cytopathology of Mycobacterial infection. Diagn Cytopathol. 2016;44(3):255–262. 10.1002/dc.23410 26800030

[7] Sfeir M, Shuetz A, Van Besien K, et al Mycobacterial spindle cell pseudotumour: Epidemiology and clinical outcomes. J Clin Path. 2018;71(7):626–630. 10.1136/jclinpath-2017-204777 29367301

[8] Hassanzad M, Valinejadi A, Darougar S, Hashemitari S, Velayati A Disseminated Bacilli-Calmette Guerin infection at a glance: A mini review of the literature. Adv Respir Med. 2019;87(4):239–242. 10.5603/ARM.2019.0040 31476012

[9] Fernandes R, Medina-Acosta E BCG-itis in two antiretroviral-treated HIV-infected infants. Int J STD and AIDS. 2010;21(9):662–663. 10.1258/ijsa.2010.010267 21097744

[10] Chen Q, Chen W, Hao F Cutaneous tuberculosis: A great imitator. Clin Dermatol. 2019;37(3):192–199. 10.1016/j.clindermatol.2019.01.008 31178102

[11] LeBoit P, Berger T, Egbert B, Beckstead J, Yen T, Stoler M Bacillary angiomatosis. The histopathology and differential diagnosis of a pseudoneoplastic infection in patients with human immunodeficiency virus disease. Am J Surg Pathol. 1989;13(11):909–920. 10.1097/00000478-198911000-00001 2802010

[12] Mosepele M, Mazo D, Cohn J Bartonella infection in immunocompromised hosts: Immunology of vascular infection and vasoproliferation. Clin Dev Immunol. 2012;2012:612809 10.1155/2012/612809 22162717 PMC3227422

[13] Balagula Y, Mattei P, Wisco O, Erdag G, Chien A The great imitator revisited: The spectrum of atypical cutaneous manifestations of secondary syphilis. Int J Dermatol. 2014;53(12):1434–1441. 10.1111/ijd.12518 25312512

[14] Rosa G, Procop G, Schold J, Piliang M Secondary syphilis in HIV positive individuals: Correlation with histopathologic findings, CD4 counts, and quantity of treponemes in microscopic sections. J Cutan Pathol. 2016;43(10):847–851. 10.1111/cup.12756 27302386

[15] Kouyoumjian S, Heijnen M, Chaabna K, et al Global population-level association between herpes simplex virus 2 prevalence and HIV prevalence. AIDS. 2018;32(10):1343–1352. 10.1097/QAD.0000000000001828 29794495 PMC5991180

[16] Kularatne R, Muller E, Maseko D, Kufa-Chakezha T, Lewis D Trends in the relative prevalence of genital ulcer disease pathogens and association with HIV infection in Johannesburg, South Africa 2007–2015. PLoS One. 2018;13(4):e0194125 10.1371/journal.pone.0194125 29617372 PMC5884493

[17] Sbidian E, Battistella M, eGoff J, et al Recalcitrant pseudotumoral anogenital herpes simplex virus type 2 in HIV infected patient: Evidence for predominant B lymphoplasmacytic infiltration and immunomodulators as effective therapeutic strategy. Clin Infect Dis. 2013;57(11):1648–1655. 10.1093/cid/cit592 24065320

[18] Yan Z, Wang L, Dennis J, Doern C, Baker J, Park J Clinical significance of isolated cytomegalovirus-infected gastrointestinal cells. Int J Surg Pathol. 2014;22(6):492–498. 10.1177/1066896914537681 24891553

[19] Dauden E, Fernandez-Buezo G, Fraga J, Cardenoso L, Garcia-Diez A Mucocutaneous presence of cytomegalovirus associated with Human Immunodeficiency Virus infection. Arch Dermatol. 2001;137(4):443–448. 11295924

[20] Mohanlal R, Karstaedt A Cytomegalovirus infection of the gastrointestinal tract in Soweto, South Africa: A look back at the clinical and histological features over 8 years. J Infect Dis Epidemiol. 2017;3:038 10.23937/2474-3658/1510038

[21] Young N, Brown K Parvovirus B19. N Engl J Med. 2004;350(6):586–597. 10.1056/NEJMra030840 14762186

[22] Molenaar-de Backer M, Russcher A, Kroes A, Koppelman M, Lanfermeijer M, Zaaijer H Detection of parvovirus B9 DNA in blood: Viruses or DNA remnants. J Clin Virol. 2016;84:19–23. 10.1016/j.jcv.2016.09.004 27664778

[23] Corcioli F, Zakrzewska K, Rinieri A, et al Tissue persistence of Parvovirus B19 Genotypes in asymptomatic persons. J Med Virol. 2008;80(11):2005–2011. 10.1002/jmv.21289 18814251

[24] Shu-hua X, Sun J Schistosoma hemozoin and its possible roles. Int J Parasitol. 2017;47(4):171–183. 10.1016/j.ijpara.2016.10.005 28012717

[25] Secor W The effects of schistosomiasis on HIV/AIDS infection, progression and transmission. Curr Opin HIV AIDS. 2012;7(3):254–259. 10.1097/COH.0b013e328351b9e3 22327410 PMC11316515

[26] Kjetland E, Leutscher P, Ndhlovu P A review of female genital schistosomiasis. Trends Parasitol. 2012;28(2):58–65. 10.1016/j.pt.2011.10.008 22245065

[27] Le L, Hsieh M Diagnosing urogenital schistosomiasis: Dealing with diminishing returns. Trends Parasitol. 2017;33(5):378–387. 10.1016/j.pt.2016.12.009 28094201

[28] Serpa J, Moran A, Goodman J, Giordano T, White A Neurocysticerosis in the HIV era: A case report and review of the literature. Am J Trop Med Hyg. 2007;77(1):113–117. 10.4269/ajtmh.2007.77.113 17620640

[29] Gyure K Infections In: Prayson R, editor Neuropathology. 2nd ed. Philadelphia, PA: Elsevier, 2012; p. 365–367.

[30] Dissanayake P, Chennuri R, Tarjan G Fine-needle aspiration diagnosis of primary hydatid disease of the thyroid; first reported case in the USA. Diagn Cytopathol. 2016;44(4):334–337. 10.1002/dc.23421 26994595

[31] Wahlers K, Menezes C, Wong M, et al Human cystic echinococcosis in South Africa. Acta Trop. 2011;120(3):179–184. 10.1016/j.actatropica.2011.08.006 21875569

[32] Von Braun, Trawinski H, Wendt S, Lubbert C Schistosoma and other relevant helminth infections in HIV-positive individuals – An overview. Trop Med Infect Dis. 2019;4(2):pii: E65 10.3390/tropicalmed4020065 31013827 PMC6631468

[33] Ramdial P, Hlatshwayo N, Singh B Strongyloides stercoralis mesenteric lymphadenopathy: Clue to the etiopathogenesis of intestinal pseudo-obstruction in HIV-infected patient. Ann Diagn Pathol. 2006;10(4):209–214. 10.1016/j.anndiagpath.2005.11.008 16844562

